# Status of kinases in Epstein-Barr virus and *Helicobacter pylori* Coinfection in gastric Cancer cells

**DOI:** 10.1186/s12885-020-07377-0

**Published:** 2020-09-29

**Authors:** Charu Sonkar, Tarun Verma, Debi Chatterji, Ajay Kumar Jain, Hem Chandra Jha

**Affiliations:** 1 The Discipline of Biosciences and Biomedical Engineering, Indian Institute of Technology Indore, Room no. 302, School Building, IIT Indore, Khandwa Road, Simrol, Indore, 453552 India; 2 Choithram Hospital and Research Centre Indore, Indore, Madhya Pradesh India

**Keywords:** Gastric cancer, *Helicobacter pylori*, Epstein Barr virus, Interleukin-2-inducible T-cell kinase, Tyrosine-protein kinase Fyn, Adenocarcinoma gastric cell

## Abstract

**Background:**

*Helicobacter pylori (H. pylori)* and Epstein - Barr virus (EBV) plays a significant role in aggressive gastric cancer (GC). The investigation of genes associated with these pathogens and host kinases may be essential to understand the early and dynamic progression of GC.

**Aim:**

The study aimed to demonstrate the coinfection of EBV and ***H. pylori*** in the AGS cells through morphological changes, expression of the kinase and the probable apoptotic pathways.

**Methods:**

Genomic DNA isolation of *H. pylori* and its characterization from clinical samples were performed. RT-qPCR of kinases was applied to scrutinize the gene expression of kinases in co-infected GC in a direct and indirect (separated through insert size 0.45 μm) *H. pylori* infection set up. Morphological changes in co-infected GC were quantified by measuring the tapering ends of gastric epithelial cells. Gene expression profiling of apoptotic genes was assessed through RT-qPCR.

**Results:**

An interleukin-2-inducible T-cell kinase (ITK) showed significant upregulation with indirect *H. pylori* infection. Moreover, Ephrin type-B receptor six precursors (EPHB6) and Tyrosine-protein kinase Fyn (FYN) showed significant upregulation with direct coinfection. The tapering ends in AGS cells were found to be extended after 12 h. A total of 24 kinase genes were selected, out of which EPHB6, ITK, FYN, and TYK2 showed high expression as early as 12 h. These kinases may lead to rapid morphological changes in co-infected gastric cells*.* Likewise, apoptotic gene expression such as APAF-1 and Bcl2 family genes such as *BAD, BID, BIK, BIM, BAX, AND BAK* were significantly down-regulated in co-infected AGS cells.

**Conclusion:**

All the experiments were performed with novel isolates of *H. pylori* isolated from central India, for the functional assessment of GC. The effect of coinfection with EBV was more profoundly observed on morphological changes in AGS cells at 12 h as quantified by measuring the tapering of ends. This study also identifies the kinase and apoptotic genes modulated in co-infected cells, through direct and indirect approaches. We report that ITK, EPHB6, TYK2, FYN kinase are enhanced, whereas apoptotic genes such as APAF-1, BIK, FASL, BAX are significantly down-regulated in AGS cells coinfected with EBV and *H. pylori*.

## Background

Cancer is the second leading cause of death globally and was responsible for an estimated 960,000 deaths in 2018. Globally, about 1 in 6 deaths occur due to cancer with gastric cancer (GC) being the third leading cause of cancer-related deaths. Despite primary management which consists of surgical resection followed by radiation therapy and chemotherapy, it is poorly prognosticated [1]. The delay in the detection of GC leads to frequent relapse and metastasis. Hence, it is imperative to find the serendipitous prognostic markers, which may be helpful in early diagnosis of GC.

The crucial link between GC and *H. pylori* is well established [2]. The *H. pylori* are considered a type I carcinogen in GC [3]. *H. pylori* is prevalent across the globe with significantly higher incidence seen in the eastern part of Asia, such as Japan and Korea [4]. *H. pylori* shows a high level of intra-species genetic diversity where strain-specific features are critical for progression of GC [4]. If *H. pylori* infection remains untreated, it colonizes the stomach and can persevere lifelong. The driving factors which turn *H. pylori* into pathogenic bacteria are poorly known. Kinases play a role as pivotal regulators in epigenetic modulation in various diseases, including cancer [5]. Recent studies suggested that *H. pylori* infection leads to the up-regulation of tyrosine kinase, MAPK cascade, PDK1, AKT3, SRC, FYN, YES, and mTOR, and dysregulation of non-receptor tyrosine kinase in cancer progression [6–9].

The viral coded protein can also cause tumorigenesis, which is derived from the transformation of the temperature-sensitive mutation of the virus [10]. Limited reports are available related to the molecular mechanism of virus mediated tumorigenesis [11]. The involvement of kinases, bacteria, and viruses in different types of cancers has been sequentially investigated for the development of cancer therapy. The protein kinase association with v-Src in-vitro was a breakthrough in the field of cancer biology [11]. Several reports suggest the association of Rous sarcoma virus with protein kinase activity related to the cancer disease [12]. Interestingly, kinases are considered as a potential target in cancer therapy.

Finally, the discovery of EBV, the first human virus associated with cancer, clearly showed the oncogenic potential of microorganisms [13]. Most of the human cancers (15–20%) are associated with a viral infection, and EBV is recognized as one of the contributors in GC (9% of all GC) [14]. The exact mechanism of EBV as an oncogenic agent in GC is poorly understood. The EBV is associated with several lymphoid and epithelial cancers and is considered as an active oncogenic agent in GC progression [15]. In the EBV associated GC, host genes such as JAK2, MET, FGFR2, BRAF, RAF, EPHA4, PAK1, PAK2, EPHB6, ERBB4, ERBB2, and ITK are up-regulated [16–22]. In contrast, FGFR4 and ROR2 genes are down-regulated in GC [23, 24]. In Asian countries, the incidence of EBV positive person developing GC are rapidly increasing (6–10% approximately). Moreover, western and central Asian countries have a considerably higher frequency of EBV positive cases [25]. Another challenging aspect is the coinfection of EBV with *H. pylori* that has been reported to cause aggressive GC [26]. Thereby, it is imperative to develop a coinfection model for investigating the progression of GC, which can be used to test the potential role of protein kinases, which is one of the hallmarks in all cancers.

To the best of our knowledge, this is the first report that shows the association of EBV and *H. pylori* (coinfection model) in GC through kinase protein. In view of above, the present study was conducted with following objectives: (1) To demonstrate the coinfection of EBV and *H. pylori* in AGS cell line for GC progression, (2) To determine the morphological changes after coinfection, (3) To evaluate the expression of the kinases in coinfection and; (4) To study the probable apoptotic pathway involved in the co-infected GC.

## Methods

### Animal cell cultures and *H. pylori* cultures

Adenocarcinoma gastric (AGS) cell line was procured from National Centre for Cell Science (NCCS), Pune, India. The cells were grown in Dulbecco’s modified Eagle’s medium (DMEM; Himedia, Mumbai, India) supplemented with 10% fetal bovine serum (FBS; BIOWEST, South America origin), 1% penicillin-streptomycin (Himedia, Mumbai, India). Infectious EBV was produced by transfection of BAC-EBV-GFPWT into HEK-293 T (human embryonic kidney cell) cells, selection followed by chemical induction. We received the transfected HEK293T EBV BAC as gift from University of Pennsylvania, USA, which were further cultured in the lab. Cultured HEK 293 T EBV BAC were induced for 5 days with 20 ng/ml tetradecanoyl phorbol acetate (TPA) and 3 mM butyric acid (Sigma-Aldrich Corp., St. Louis, MO). The supernatant from cell culture was collected and treated with DNAse. The viruses were concentrated by ultracentrifugation 23,500×g at 4 °C for 1 h 30 min and quantified through qRT-PCR [27–29]. The infective dose of EBV was determined by infecting 25 × 10^4^ AGS cells seeded in 6 well plates with 0, 25, 50, 75, 100, and 125 μl of the isolated virus. It was followed by isolation of mRNA, preparation of cDNA, and RT-qPCR for detection of EBNA-1. EBNA1 oncoprotein is the only viral protein expressed in all forms of latency during EBV infection [30]. We confirmed the presence of EBV in the AGS cells through RT-qPCR. RT-qPCR is a recognized method for determining multiplicity of infection which has been used in other studies as well, and thus we used this method to determine the titer value [31, 32]. We found that the infective dose resulting in high expression of EBNA-1 was 100 μl which corresponds to 20 MOI [33, 34]. The *H. pylori* strain I10 was kindly provided by Dr. Asish Kumar Mukhopadhyay (National Institute of Cholera and Enteric Diseases, ICMR, Kolkata, India). The reference strain I10 has also been used and reported in one of our previous studies [35]. The biopsy sample and gastric juice were provided by Dr. Ajay Kumar Jain (gastroenterologist), Choithram Hospital and Research Centre, Madhya Pradesh, India. Cell culture experiments were performed at 37 °C in a humidified environment supplemented with 5% CO_2_. The *H. pylori* strains were grown in Whitley DG250 Anaerobic Workstation at 37 °C with micro-aerophilic conditions (85% N_2_, 10% CO_2_, and 5% O_2_).

### Isolation and identification of *H. pylori*

The rapid urease test kit (CLO Test Ballard Medical Products, Draper, UT, USA) was used on gastric biopsy samples to check the presence of *H. pylori*. Gastric juices were also obtained from RUT positive patients. The tissue homogenate was prepared by crushing the biopsy samples. A loop-full of tissue homogenate was cultured on a non-selective medium such as Brain Heart Infusion media (BD-DIFCO, USA). This media was supplemented with *H. pylori* selective antibiotics such as vancomycin, cefsulodin, amphotericin, and trimethoprim with 10, 5, 5, and 5 mg/L, respectively (Sigma Aldrich, St Louis, MO, USA). These plates were incubated in a micro-aerophilic environment for 3–5 days. A point sized colony was identified and used for the Gram staining. The colony was screened through morphological similarities with *H. pylori*. The samples were human gastric biopsy and gastric juice; thus, they were named HB and HJ, respectively, followed by a numeral that denotes the patient number according to the sequence of sample collection.

Further, *H. pylori* were confirmed by polymerase chain reaction (PCR) for 16S rRNA gene. For the present study, two clinically isolated bacterial strains were considered; HB1 (human biopsy sample#1), HJ9 (Human gastric juice sample#9), and one reference strain I10. The *H. pylori* bacteria were grown in selective media in a 14 ml round bottom snap cap tube (14 ml round bottom snap cap tube (BD Biosciences, Franklin Lakes, NJ, USA - Catalogue No. 352001). They were then incubated in the microaerophilic chamber for 72 h. Subsequently, 150 μl of grown culture was placed in duplicate in 96 well flat-bottom plates, and optical density (OD) was recorded at 600 nm (Synergy H1 Hybrid Multi-Mode Reader, BioTek). An optical density of 0.3 at 600 nm represents 500 million CFU/ mL [36]. The final OD value was normalized with media as a negative control [35, 37]. The number of bacterial cells per ml (CFU/mL) of culture was evaluated according to the final OD, and the required volume of the bacterial culture for infection was then calculated.

### Cell proliferation measurement

25 × 10^4^ AGS cells were infected with *H. pylori* isolate I10, HB1, and HJ9 at MOI 100 and incubated at 37 °C for 6–8 h, subsequently further infected with EBV at an infective dose of 100 μL. The AGS cells were infected with EBV, EBV-I10, EBV-HB1, and EBV-HJ9, respectively. Uninfected AGS cells were used as control. The cells were trypsinized and diluted with trypan blue (1:1). The cells were counted through hemocytometer at 6, 12, 24, and 36 h, respectively, with white cells being viable and blue depicting non-viable cells [26]. The morphological changes in the cells were evaluated by DAPI staining. The confocal laser scanning microscopy (CLSM) was performed using Multi-photon laser (FV1200MPE, IX83 Model, Olympus). The ImageJ software was used for the cell length measurements. All the statistical data was analyzed by GraphPad Prism software.

### Coinfection of EBV and *H. pylori*

**Fig. 1:**
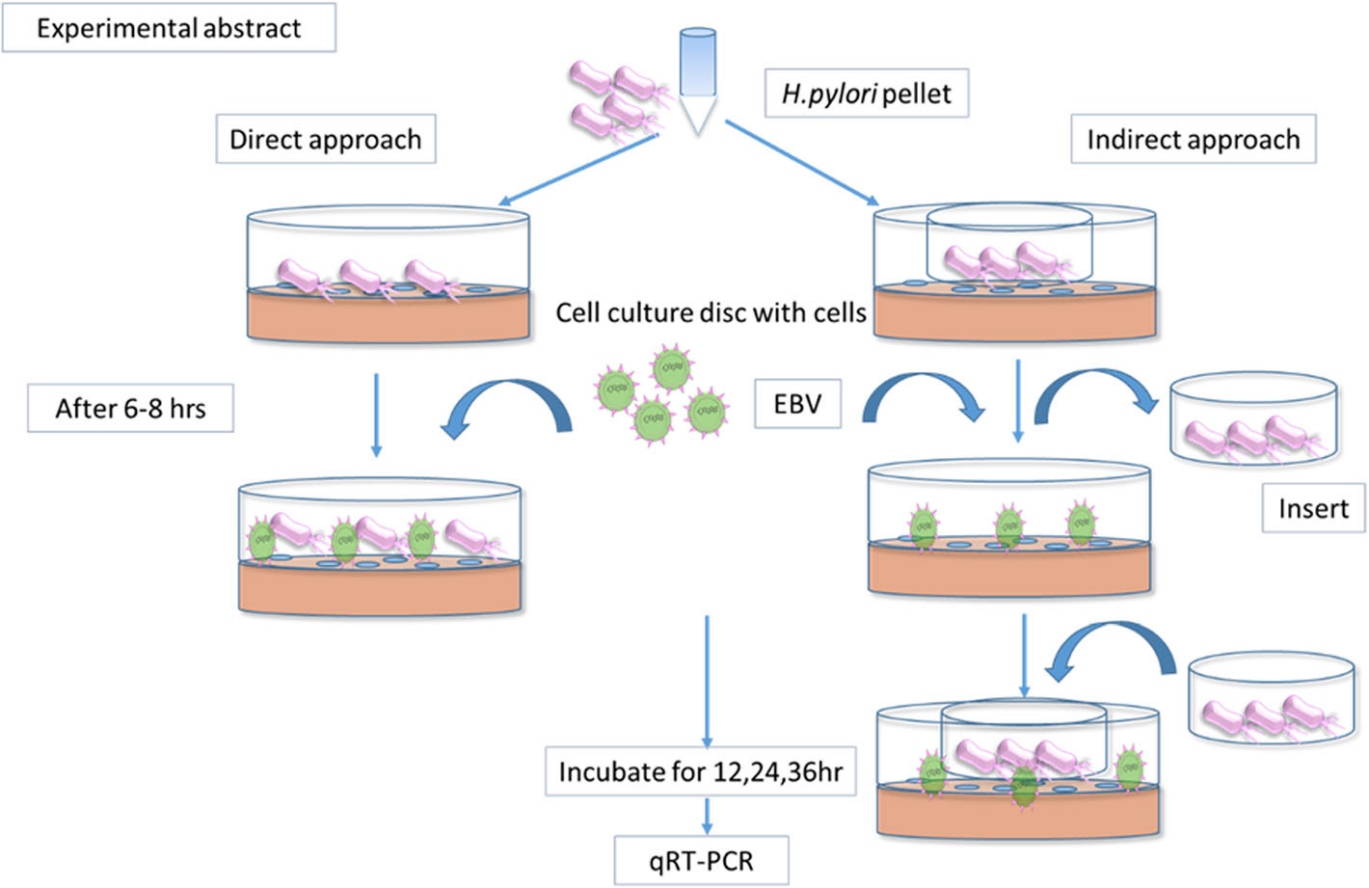
Schematic representation of the experimental setup performed in the report. Two approaches used for an experiment wherein Direct approach *H. pylori* was added to cells at MOI 100 without an insert. After 6–8 h, these cells were infected with EBV. Whereas in the Indirect approach, *H. pylori*-infected the cultured cells through an insert. Again after 6–8 h insert was removed, cells were infected with EBV, and again, the insert containing bacteria resumed in its original position

AGS cells (25 × 10^4^) were seeded in 6 well plates followed by *H. pylori* infection through transwell inserts of 0.45 μm at MOI of 100. After 6–8 h, transwell were removed, and the cells were infected with EBV at an infective dose of 100 μl. This was followed by centrifugation at 2000 rpm for 20 min followed by re-insertion of transwell insert. This setup was then incubated for various time intervals. For the direct infection approach, the rest of the protocol remained the same without the use of a transwell. The graphical representation of the experiments performed in the project is shown in Fig. 1, which depicts the procedure of direct and indirect coinfection in the cells, which were followed by other experiments like RT-qPCR. After 12, 24, and 36 h, cells were scrapped through cell lifter and centrifuged at 3000 rpm for 5 min to get the pellet. This pellet was washed with phosphate buffer saline (PBS; pH 7.4; 10 mM) twice and stored at − 20°c till further use.

### Quantitative RT-PCR

The cells were infected with the bacteria followed by virus incubation for 12 and 24 h, respectively. The Trizol reagent was used for RNA extraction from co-infected cells. The isolated RNA (0.5 μg) was used to synthesize the cDNA with the help of the Takara cDNA synthesis kit. The primers for kinase genes are listed in (Table 1), subsequently primers for apoptotic genes and for *H. pylori* are mentioned in (Table 2). The thermocycler conditions used for the real-time PCR (RT-qPCR) were for 40 cycles and set at 94 °C for 15 s, 54 °C for 20 s, and 72 °C for 20 s, respectively, using SYBR green. The expression analysis was carried out using 2^-ΔΔt^ method.

**Table 1:** list of primers used with their primer sequences

S.No.	Gene name	Forward primer	Reverse primer
1.	EPHB6	ATGAAGTGCCCTCTGCTGTC	CTGCCTGGTCATAGTAGCGG
2.	MAPK1	AACAGGCCCATCTTTCCAGG	CCAGAGCTTTGGAGTCAGCA
3.	SRC	ACATCCCCAGCAACTACGTG	CAGTAGGCACCTTTCGTGGT
4.	AKT3	ACCGCACACGTTTCTATGGT	TTCATGGTGGCTGCATCTGT
5.	JAK2	TGGGGTTTTCTGGTGCCTTT	TAGAGGGTCATACCGGCACA
6.	PAK1	ACAGGAGGTGGCCATTAAGC	CACAGCTGCAATTTGGCCTT
7.	PAK2	ATTGGACAAGGGGCTTCTGG	CCACATCAGTGAGTGACCCC
8.	ERBB2	CGCTGAACAATACCACCCCT	GCCAGCTGGTTGTTCTTGTG
9.	FGFR2	CCAACTGCACCAACGAACTG	ACTGTTCGAGAGGTTGGCTG
10.	METMET	GTCCTGCAGTCAATGCCTCT	GTCAGCCTTGTCCCTCCTTC
11.	PDK1	AAGTTCATGTCACGCTGGGT	GCATCTGTCCCGTAACCCTC
12.	ROR2	ACGTACGCATGGAACTGTGT	CGGCACATGCAAACCAAGAA
13.	ERBB4	ACAGGGGGCAAACAGTTTCA	AGCCCACCAATTACTCCAGC
14.	FYN	CTCAGCACTACCCCAGCTTC	AGGTCCCCGTATGAGACGAA
15.	ITK	ATTATCTACGCACCCAGCGG	ATGCCCTCACACACATCCAG
16.	TYK2	CCCATGGCTTGGAAGATGGT	ACTCAGCTTGATGAAGGGGC
17.	YES 1	GCTCCTGAAGCTGCACTGTA	GCATCCTGTATCCTCGCTCC
18.	EPHA4	AAGGCTATCGGTTACCCCCT	CTTCAAGCTGTTGGGGTTGC
19.	MERTK	GCCCCATCAGTAGCACCTTT	TGCACGTAGCATTGTGGACT
20.	TYRO3	CAAACTGCCTGTCAAGTGGC	CCCGCCAATGAGGTAGTTGT
21.	BRAF	AGAGGCGTCCTTAGCAGAGA	ATCGGTCTCGTTGCCCAAAT
22.	MTOR	TCGCTGAAGTCACACAGACC	CTTTGGCATATGCTCGGCAC
23.	RAF1	AATCAGCCTCACCTTCAGCC	AAAGAGCCTGACCCAATCCG
24.	FGFR4	GAGTCTCGTGATGGAGAGCG	AGTTATAGCGGATGCTGCCC

**Table 2:** List of primers for apoptotic genes

S.No.	Gene name	Forward Primer	Reverse Primer
1.	PARP1	GGCGATCTTGGACCGAGTAG	AGCTTCCCGAGAGTCAGGAT
2.	APAF1	CTTGCTGCCCTTCTCCATGA	TTGCGAAGCATCAGAATGCG
3.	FASR	CCTGCCAAGAAGGGAAGGAG	TTTGGTGCAAGGGTCACAGT
4.	BID	CTGCAGGCCTACCCTAGAGA	GTGTGACTGGCCACCTTCTT
5.	BIK	ACCTGGACCCTATGGAGGAC	CTGAGGCTCACGTCCATCTC
6.	BIM	CTTCCATGAGGCAGGATGAA	TCCAATACGCCGCAACYCYY
7.	BAX	CATGGGCTGGACATTGGACT	AAAGATGGTCACGGTCTGCC
8.	NOXA	CAAGAACGCTCAACCGAGCC	GCCGGAAGTTCAGTTTGTCTC
9.	FAS	GGACCCTCCTACCTCTGGTT	GCCACCCCAAGTTAGATCTGG
10.	FADD	CACCAAGATCGACAGCATCG	AGATTCTCAGTGACTCCCGC
11.	BAK	GGTTTTCCGCAGCTACGTTT	TAGCGTCGGTTGATGTCGTC
12.	CASPAS9	TGCTCAGACCAGAGATTCGC	TCTTTCTGCTCGACATCACCAA
13.	16s RNA (*H.pylori*)	CTGGAGAGACTAAGCCCTCC	ATTACTGACGCTGATTGCGC

### Statistical analysis

The analysis and quantification of the experimental setup were done through Image J and Graph Pad Prism software version 6, respectively. Biological triplicate was required for each experiment. Quantitative data were shown as mean ± SD. The difference comparison between groups was analyzed with independent t-test or ANOVA. In all analyses, *p* < 0.05 was seen as a significant level.

## Results

### Isolation and characterization of *H. pylori* isolates

We have successfully extracted five isolates of *H. pylori* from gastric biopsy and gastric juices*,* namely, HB1, HB10, HJ1, HJ9, and HB14, which was followed by Gram staining, where I10 was used as reference strain (Fig. S1). Genomic DNA isolation was performed and 16sRNA primer was used for the screening of the bacteria along with reference strain (Fig. S2). Among the five isolates, two isolates were selected for further experiments. The reference strain I10, along with two isolated strains of the *H. pylori* isolates HB1 and HJ9, were used for further experiments. The PCR was performed using *H. pylori* strains’ genomic DNA as template to amplify 16 s rRNA genes, with a product size of 110 bp (Fig. S3).

### *H. pylori* and EBV coinfection leads to morphological changes

**Fig. 2:**
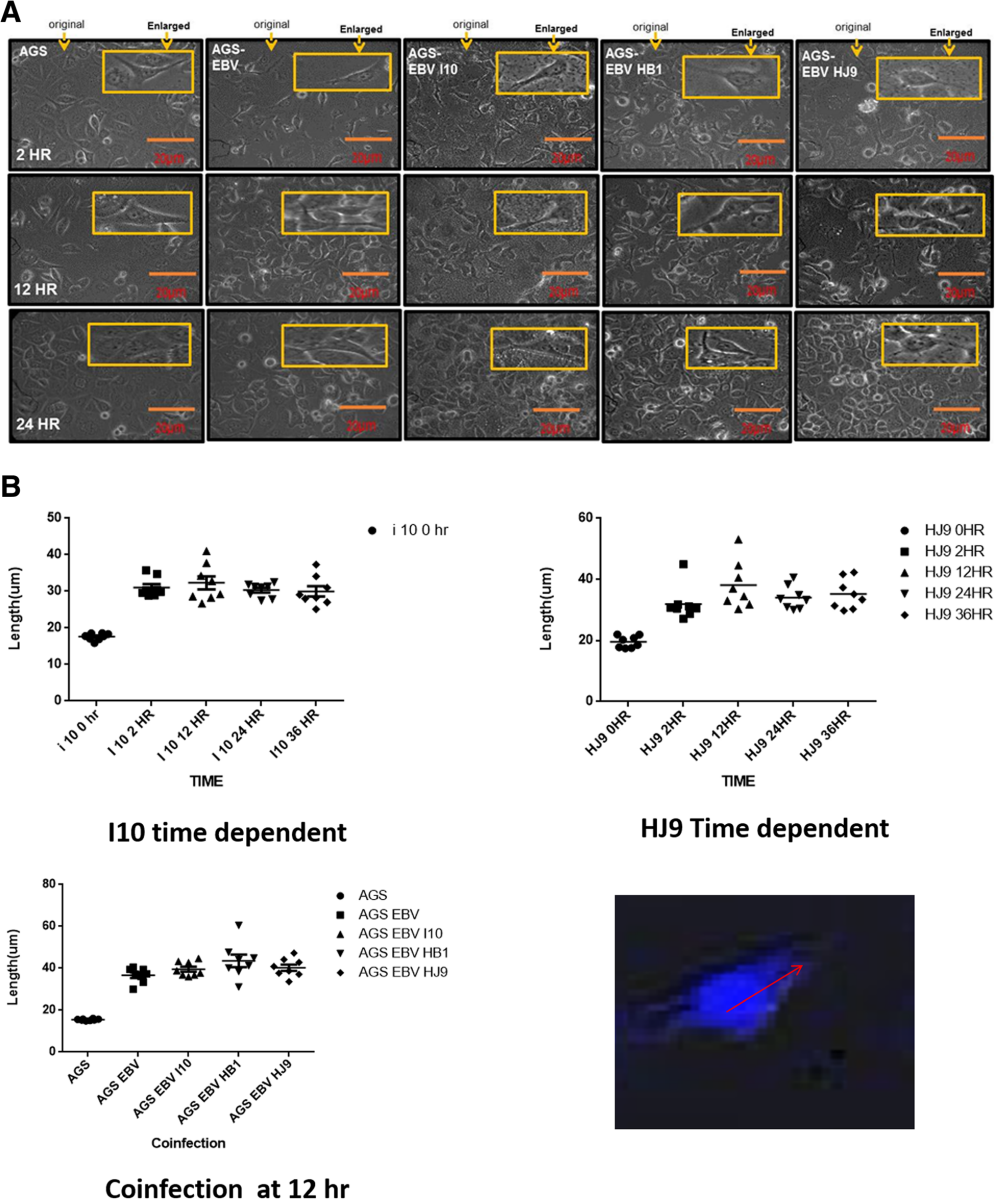
*H. pylori* and EBV coinfection lead to morphological changes. **a** AGS cells were infected with EBV, and then AGS cells were infected with EBV and *H. pylori* I10, HB1, HJ9, respectively. Changes in the number of cells and morphological changes were observed at 2 h, 12 h, and 24 h where insert image shows the enlarged image of morphological changes**. b** Quantification of extended length was done for all experiments through Graph Pad Prism software. The single-cell DAPI stained picture of the cell determines the way the cell length is measured through the software. The arrow depicts the measurement of the cell length

Previous studies have shown that morphological and phenotypic changes can be detected in virus-infected cells [38]. These cells acquire a characteristic elongated cell shape with an invasive phenotype that contributes to tumor invasion and metastasis [39]. Our data shows that similar morphological changes such as elongated tapering ends were observed in AGS infected *H. pylori* at 24 h [40]. Our results show that tapering ends of co-infected cells were found to be more elongated as compared to those seen in uninfected cells (Fig. 2a, S4). Interestingly, the co-infected AGS showed morphological changes even after 12 h of incubation, which may reflect the positive synergistic effect of EBV and *H. pylori* in cell proliferation.

The elongations in tapering ends were quantified by confocal laser scanning microscopy using DAPI stain at various time intervals such as 12, 24, 36, and 48 h, respectively (Fig. S5). The analysis and quantification of the experimental setup were done through Image J and Graph Pad Prism software, respectively (Fig. 2b). Our results asseverate that the maximum length projection appears at 12 h post-infection (Fig. S6). The lengths of the sharp end of co-infected cells were measured to estimate the effect of EBV on AGS cells at 12 h incubation. Our data shows that approximately 10 fold increase in the tapering end length was observed in comparison with the control, which suggests that EBV has a positive effect on cell growth and migration (Fig. 2b).

### Effect of coinfection on cell proliferation

**Fig. 3:**
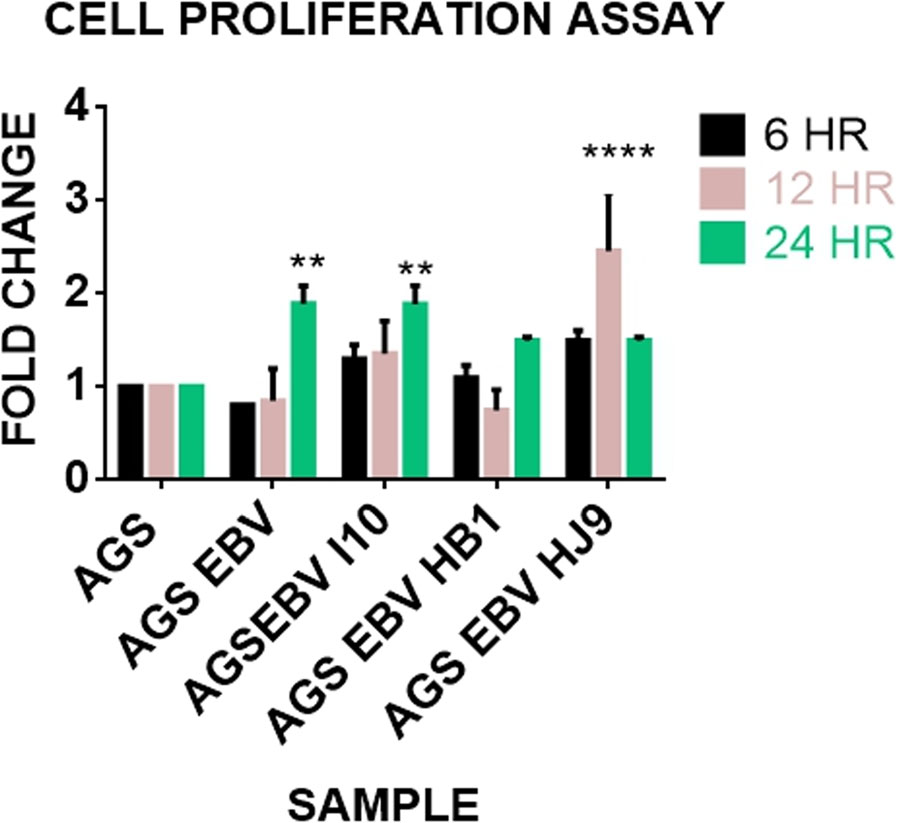
Effect of coinfection in cell proliferation. Cell proliferation of AGS when treated with EBV and AGS-EBV with different strains of bacteria I10, HB1, and HJ9, respectively, at different time points of 6, 12, and 24 h

Previous studies have suggested that EBV and *H. pylori* both may promote cell proliferation through inducing morphological changes [41, 42]. At 12 h the cell proliferation assay shows a decrease in cell number when cells were infected with EBV alone. However, an increase in cell proliferation is observed when EBV infection is followed by a bacterial infection (Fig. 3). Hence, our finding suggests that bacteria may provide positive thrust for cell proliferation. In comparison with control, the cells co-infected with HJ9 showed an approximately 2 fold increase in cell proliferation at 12 h. However, in HB1 co-infected cells, no significant change in proliferation was observed till 24 h. The positive effect of bacterial co-infection on the growth of cells is strain dependent, and it can affect the proliferation in a time-dependent manner. Interestingly, the cell number increases significantly when AGS cells infected with EBV alone or co-infected with *H. pylori* and EBV, when compared to un-infected AGS cells.

### Assessment of kinase expression through a secretory and adhesive mechanism of bacteria

To evaluate differential expressions of several kinases, both direct and indirect infection methods were used [27]. In the indirect approach, the effect of proteins secreted from bacteria was assessed; and in the direct approach the kinases that are mostly affected by adherence were evaluated. Here, we tried to investigate the kinases of secretory and adherence pathways of *H. pylori* to get an insight into the underlying strategy which involves the cooperation of *H. pylori* in EBV-driven proliferation of gastric epithelial cells. Hence, already developed *H. pylori* and EBV coinfection model was used for AGS human gastric epithelial cells. This model would give us access to investigate the effect of molecules secreted by *H. pylori*. Hence we used the 0.45 μm insert, which has been used for a similar purpose in the previous reports [27, 43]. As the effect of adherence of *H. pylori* to gastric mucosa through CagA is linked to the severity of gastritis, it was intriguing to compare the effect of secretory proteins and adherence of bacteria in gastric cells infected with EBV [44–46].

**Fig. 4:**
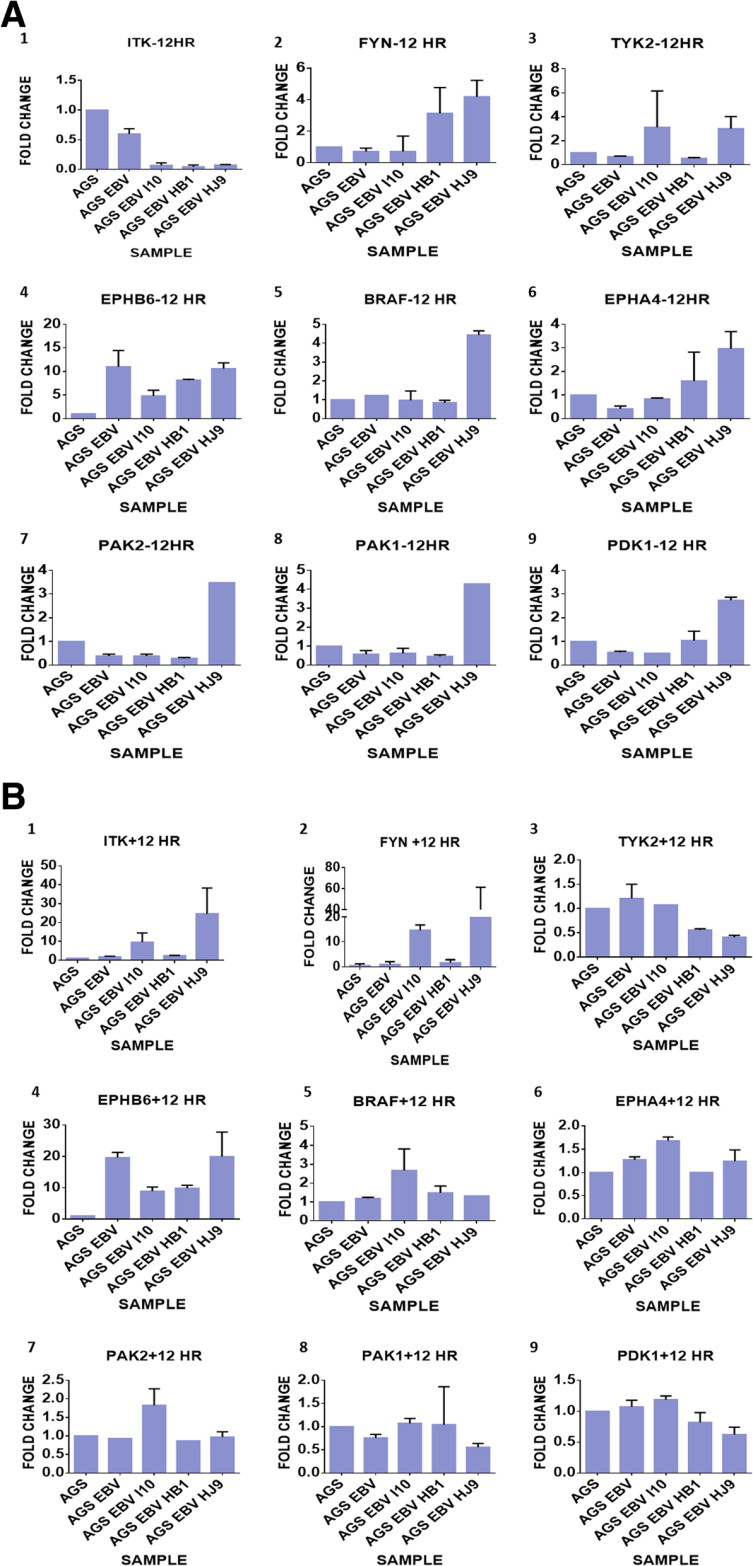
Assessment of kinase expression through the secretory and adhesive mechanism of bacteria. Gene’s expression was shown with a direct and indirect approach at different time points. **a**, **b** at 12 h. Where “+” indicated experiment performed with insert, i.e., indirect approach and “-” indicates experiment performed without an insert, i.e., direct approach

All 24 kinases were screened based on their presence in GC either infected with *H. pylori* or EBV alone. Their gene expressions were evaluated at a time interval of 12, 24, and 36 h, respectively. Out of 24 genes, eight genes showed considerable changes in gene expression, which are BRAF1, ITK, TYK2, FYN, PAK1, PAK2, PDK1, and EPHB6. Among the eight genes, four genes showed significant changes in expression, which were ITK, FYN, TYK2, and EPHB6. Reports suggest a high expression of ITK, FYN, and TYK2 in GC, whereas EPHB6 showed reduced expression in GC [47, 48]. According to our experimental data, TYK2 and EPHB6 transcripts were enhanced by the indirect coinfection approach, whereas the other two genes, like FYN and ITK, were observed to be up-regulated in the direct coinfection approach (Fig. 4).

In the direct approach at 12 h incubation, ITK was found to be significantly down-regulated in AGS cells co-infected with EBV-I10, EBV-HB1, and EBV-HJ9 compared to controls at 12 h time point. Interestingly, ITK was slightly down-regulated in AGS-EBV compared to control AGS cells. However, there is a slight down-regulation of the ITK gene in AGS-EBV infected cells in comparison with AGS (Fig. 4A.1). Additionally, the FYN gene transcript showed non-significant changes in AGS-EBV and EBV-I10. However, FYN levels were considerably up-regulated in EBV-HB1 and EBV-HJ9 (Fig. 4A.2). Noticeably, FYN expression was 2.5-fold higher. Hence, in comparison to AGS within EBV-I10, EBV-HB1 and EBV-HJ9 showed about 2.5-fold increases in expression in FYN when compared to controls. Further, the TYK2 gene transcript showed down-regulation in AGS-EBV and EBV-HB1 while showed enhanced up-regulation in EBV-I10 and EBV-HJ9 (Fig. 4A.3). The EPHB6 gene transcript showed more than 6-fold was up-regulated by AGS-EBV and EBV-HB1, while, coinfection group such as EBV-I10 and EBV-HJ9 showed more than 2.5-fold and 10-fold enhanced expression, respectively (Fig. 4A.4). Therefore, based on the gene expression profiling, it is clear that TYK2 and EPHB6 may have a pivotal role in early prognosis and pathway determination.

Furthermore, at 24 h time point, ITK expression does not vary significantly in AGS-EBV and EBV-HB1, while considerable down-regulation was observed showing a mild and significant decrease in expression in EBV-I10 and EBV-HJ9, respectively (Fig. S7.1). However, FYN expression showed a 2.5 to a 60,000-fold increase in expression of AGS-EBV and EBV-HJ9, respectively. Additionally, EBV-HB1 showed a slight increase in expression, whereas EBV-I10 showed no remarkable changes in gene expression in comparison to AGS (Fig. S7.2). The TYK2 expression was significantly reduced in both EBV-I10 and EBV-HJ9 while showing no noticeable changes in AGS-EBV and EBV-HB1 (Fig. S7.3). The EPHB6 expression level was found to be detected mildly and significantly less in EBV-HB1 and EBV-HJ9, respectively, while no changes were observed in the expression of EPHB6 in AGS-EBV and EBV-I10 respectively (Fig. S7.4). Importantly, there were no significant changes observed at 36 h in these cells (Fig. S8).

In the indirect approach at 12 h, ITK and FYN expression were significantly increased from about 6–10 fold and 10–50 folds in EBV-I10 and EBV-HJ9, respectively (Fig. 4B.1, B.2). In comparison, these genes showed no significant changes in AGS-EBV and EBV-I10. However, the TYK2 gene showed a significant decrease in the expression of EBV-HJ9 and EBV-HB1, while no considerable changes were observed in AGS-EBV and EBV-I10 (Fig. 4B.3). The EPHB6 gene transcript showed a more than 10fold increase in AGS-EBV and EBV-HJ9, and more than 6 fold increases in EBV-I10 and EBV-HB1 (Fig. 4B.4). ITK gene expression at 24 h showed more than 10,000 to 50,000 fold increase in EBV-HB1 and AGS-EBV, respectively, whereas 20 fold increase is observed in EBV-HJ9 (Fig. S9.1). In the FYN gene, no significant changes were observed in AGS-EBV, while more than 2.5, 10, and 20 fold increase in expression were observed in EBV-HJ9, EBV-I10, and EBV–HJ9, respectively (Fig. S9.2). The TYK2 gene expression decreased mildly, in AGS-EBV, and no changes were found in EBV-I10, while a significant increase is observed in EBV-HB1 and EBV-HJ9 (Fig. S9.3). EPHB6 gene showed approx. 20 fold increase in the expression in of AGS-EBV and approx. 2.5 fold increase in EBV- HJ9, EBV-I10, and EBV-HB1, respectively (Fig. S9.4). In 36 h, the ITK gene showed more than 6, 10, 250 fold increase in AGS-EBV, EBV-I10, EBV-HB1, respectively, and a significant decrease in EBV-HJ9 (Fig. S10.1). The FYN gene transcript showed 17,000, 20, 250, 4 fold increased expression in AGS-EBV, EBV-I10, EBV-HB1, and EBV-HJ9, respectively (Fig. S10.2). The TYK2 gene showed no significant changes in any sample (Fig. S10.3). EPHB6 gene showed an increase in expression of the transcript, with 6, 17, 28, and 30 fold in AGS-EBV, EBV-10, EBV-HB1, and EBV-HJ9, respectively (Fig. S10.4). Hence, our findings suggest that two or more mechanisms may be involved in these experiments.

### Investigation of apoptotic markers in co-infected gastric epithelial cell lines

**Fig. 5:**
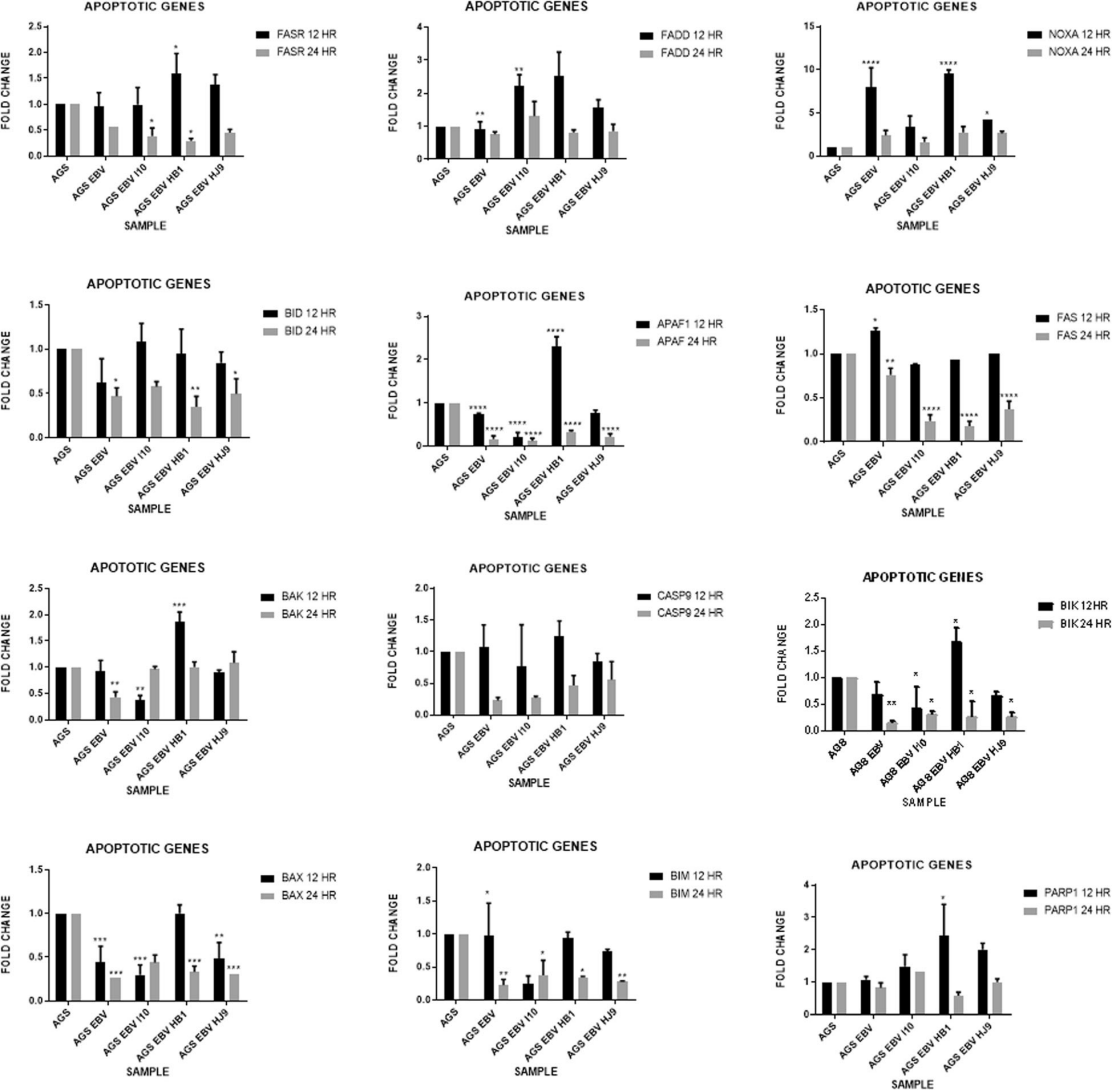
Investigation of apoptotic markers in co-infected gastric epithelial cell lines: Gene expression expressions of apoptotic genes were assessed at 12 and 24 h apoptosis after coinfection of AGS with bacteria and virus. The results are shown as the mean SD of three independent experiments where *p < 0.05, **p < 0.001, ****p < 0.0001 analyzed through two-way ANOVA

It is well reported that apoptotic genes are altered with *H. pylori* and EBV infection in gastric epithelial cell lines individually; however, studies on effect of coinfection on apoptotic genes have been modest [49]. Therefore, to identify the apoptotic genes crucial during coinfection, twelve apoptotic genes were studied that were specific for GC, whose primers have been listed in (Table 2) [50]. Their expression levels were evaluated at a time interval of 12 and 24 h of incubation. However, to determine the early apoptotic marker, 12 h was chosen as a time point to proceed with the further investigation of gene expression. The apoptotic genes such as APAF, BIK, FASL, and BAX were found to be significantly down-regulated at 24 h, which implies their potential role in cell proliferation (Fig. 5).

## Discussion

*H. pylori* usually infect during childhood, where its site of residence is the stomach for decades, causing GC, peptic ulcer, and gastritis. This bacteria is known to infect half of the world population [51].

This study includes *H. pylori* isolates from central India for the detection of the early development of GC. Consistent with previous reports, morphological changes were observed due to bacterial infection, which supports aggressive cell proliferation [52]. CagA + *H. pylori* infection in AGS cells causes a hummingbird phenotype by dephosphorylation of vinculin. Hence, vinculin may be one of the reasons for the morphological changes [53]. But as the coinfection resulted in a different morphology, there might be other gene involvement as well. This study demonstrates morphological changes in AGS cells infected with bacteria followed by EBV co-infection. In the co-infected cells, the invasive form was observed at 12 h compared to a previous study in which they were observed at 24 h [54]. These morphological changes may be associated with the possible role of EBV and *H. pylori* co-infection in early cell transformation in the gastric epithelial cell line.

Further, we were able to quantify the tapering ends by infecting the cells with bacteria at different time intervals. After 12 h incubation of co-infected cells, a remarkable elongation of tapering ends of cells was observed.

In this study, 12 h seems to be a potential time interval to evaluate the effect of EBV on the cells infected with bacteria. These co-cultured AGS cells with *H. pylori* strains and EBV showed an increased number of hummingbird-like cells. This phenotype is considered to promote scattering and spreading of cells, which may be important in carcinogenesis [50, 55]. But to the best of our knowledge, no such study for length quantification has been done previously for this purpose.

As per our knowledge, this is the first report to demonstrate the differential expression of kinase in co-infected cells using two different approaches, i.e., with transwell inserts (indirect approach) and without transwell (direct approach). With indirect and direct approaches, we aimed to identify the most affected kinase at various time intervals of 12, 24, and 36 h, respectively. The TYK2 and EPHB6 gene were found to be up-regulated by adherence of bacteria to the cells in the presence of EBV, whereas secretory proteins of bacteria up-regulate ITK and FYN expression in the presence of EBV. Though, the expression of genes varies with infection of AGS with EBV, EBV-I10, EBV-HB1, and EBV-HJ9, respectively. Moreover, in the direct approach, the ITK gene showed similar down-regulation in the coinfection of AGS with EBV- I10, EBV-HB1, and EBV-HJ9. In contrast, the TYK2 gene showed significant upregulation in comparison to I10 and HJ9 co-infected cells than in infection with EBV or EBV-HB1 only. In 12 h, EPHB6 gene transcript also showed a significant increase in the expression in all co-infected cells. However, the EPHB6 gene showed the highest expression in EBV-HJ9 co-infected cells. Similar results were observed with the FYN gene at 24 h. Hence, our findings suggest that TYK2, EPHB6, and FYN can be used for an early prognosis for GC. With the Indirect approach, the ITK gene showed a remarkably significant increase in all infection. In contrast, cells infected with EBV alone showed the highest expression, in comparison to EBV-I10, EBV-HB1, and EBV-HJ9 whose expression were significantly increased. An earlier study conducted on breast cancer cells (MCF-7) found that FYN gene expression was higher at 24 h [56]. Moreover, the expression of TYK2 and ITK was increased in gastric tissue samples [57]. EphB6, an Eph receptor that doesn’t have tyrosine kinase activity, was reported to be expressed in some human cancers. Ephb6 with APC mutation is found to be overexpressed in colorectal cancer [16]. Also, reports have suggested that these kinases may have a role in gastric cancer progression [58].

A similar trend was observed in the FYN gene with the exception of expression in EBV-HJ9, which was reduced in comparison with EBV-I10. Contrarily, in EPHB6, considerable up-regulation was observed in all cases. Hence, when the secretory pathway of *H. pylori* is concerned, ITK, FYN, and EPHB6 can be investigated thoroughly for further studies.

Moreover, *H. pylori* consist of various genes that contribute to enhancing its infection, such as T4SS-pilus localized protein CagA, vacuolation causing secretory protein VacA and outer membrane protein BabA. CagA+ *H. pylori* strain increases the risk of distal GC as it uses the integrin receptor present on the host’s cells for its entry in the cells [59]. CagA bridges the T4SS to integrin α5 β1 on host cells, which activates the SRC and focal adhesion kinase, which ensures that CagA is phosphorylated at the site of infection [40]. VacA is a secretory protein that causes vacuolation in cultured epithelial cells. VacA binds to integrin β2 and blocks interleukin-2 mediated signaling, which causes downregulation of the Ca2 + −dependent phosphatase calcineurin and inhibits antigen-dependent proliferation of transformed T cells [60]. Eventually, *H. pylori* interfere with tyrosine kinase, Crk, GTPase, and MAP kinase signaling leading to peptic ulcer, gastritis, and GC [61]. Although the site of the residence of *H. pylori* remains to be within the semi-permeable mucous gel layer of stomach facing towards the apical surface of gastric epithelial cells, about 20% of the bacteria is known to bind with the epithelium [62]. When genome analysis of *H. pylori* strains was done, a very high proportion of protein-encoding for the open reading frame was identified in the outer and inner membrane of bacterium which is known as outer membrane proteins (omPs) such as BabA which has a role in increased mucosal inflammation, atrophy and severe gastric injury [63, 64].

**Fig. 6:**
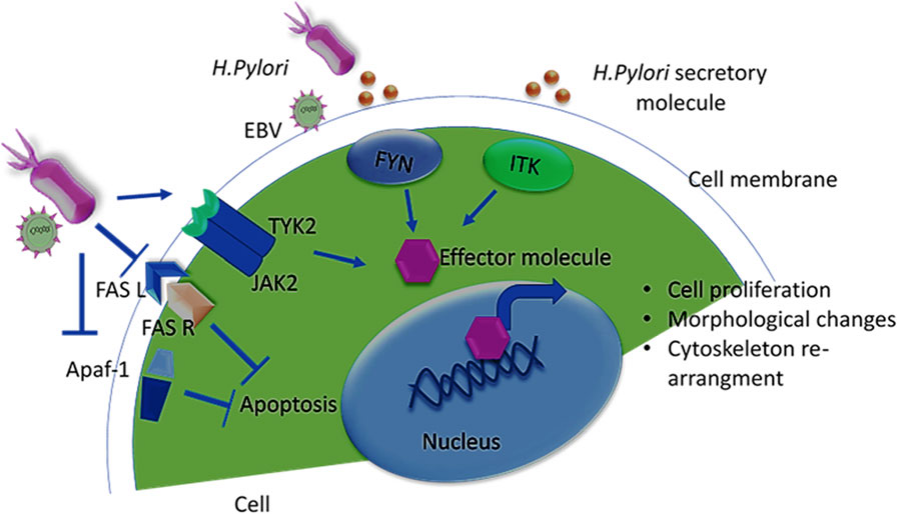
Graphical abstract of the experimental outcome in the report. *H. pylori* secretory molecule activates two kinases FYN and ITK, which may activate the effector molecule leading to the nucleus for cell proliferation, morphological changes, and cytoskeleton rearrangement. *H. pylori* adherence activates TYK2 kinase and inhibits apoptotic genes like FASL, FASR, and APAF-1, which contribute to cell proliferation

Importantly, apoptosis is a regulatory action taken by the cell for cell replacement and damaged cell removal, which can be characterized by chromatin condensation, cell shrinkage, and formation of apoptotic bodies [65]. This process is the result of the extrinsic pathway (extracellular stress) and the intrinsic pathway (intracellular stress) [66]. The death receptor is located at the cell surface, such as Fas/Fas ligand, and is induced by extracellular stress. In comparison, the intrinsic pathway is induced mainly through intracellular stress, which is associated with mitochondria, for example, APAF-1 and Bcl2 family [67]. To explore the expression of apoptotic genes through the direct approach, we selected nine apoptotic genes that have been associated with GC. Our experiment found that apoptotic genes, namely APAF-1, BIK, FASL, and BAX, were significantly down-regulated at 24 h (Fig. 6). Earlier reports suggested that apoptotic genes like APAF-1, Bcl-2, BAX, and Bcl-2 family were found to be up-regulated in gastric cancer tissues [50, 67]. Experiments performed with *H. pylori* in epithelial cell background also demonstrated the expressional differences for APAF-1, Fas-Fas ligand, and Bcl-2 related genes (Bcl-2, BAX, and BAK) genes at 48 h [68]. Furthermore, based on the experiments performed in the report, a comprehensive representation of the outcome of experiments is diagrammatically illustrated in Fig. 6, where the effect of direct and indirect coinfection in kinase and apoptosis-related signaling pathway is diagrammatically represented.

## Conclusion

Our study uses clinical *H. pylori* isolates along with reference strain to find its phenomenal changes in gastric epithelial cells along with EBV. The remarkable effect of coinfection on morphological changes found to be in 12 h intervals on implementing quantification of tapering ends. This study also demonstrated the kinase and apoptotic genes that might be affected in co-infected cells through direct and indirect approaches. Where ITK, EPHB6, TYK2, and FYN kinase are highly expressed kinase genes and APAF, BIK, FASL, and BAX are the significantly down-regulated apoptotic genes. ITK and TYK2 are receptor tyrosine kinase, which is specifically involved in cellular differentiation, survival, and proliferation and contains the conserved domain of Ig-domains. In stark contrast, non-receptor tyrosine kinase-like FYN, essential for enzyme regulation and substrate identification, was found to be up-regulated by direct dual infection. Hence, their downstream interlinked pathway can provide a potential strategy to understand the progression of GC. However, we do consider the fact that the number of strains used that were isolated from the patients is limited, and further investigation is required for drawing a more consequential conclusion.

## Supplementary information

**Additional file 1.**

## Data Availability

All-important data are presented in the manuscript or supplementary figures. Some other supporting information that may not be crucial or affecting result interpretation is not included. Moreover, these data can be available from the corresponding author on a reasonable request.

## Abbreviations

GC: Gastric cancer
*H. pylori*: *Helicobacter pylori*
EBV: Epstein barr virus
BRAF1: B-Raf proto-oncogene, serine/threonine kinase
ITK: Interleukin-2-inducible T-cell kinase
TYK2: Tyrosine kinase 2
FYN: Tyrosine-protein kinase Fyn
PAK1: P21 protein (Cdc42/Rac)-activated kinase 1
PAK2: P21 protein (Cdc42/Rac)-activated kinase 2
PDK1: Pyruvate dehydrogenase kinase1
EPHA4: Ephrin type-A receptor 4 precursor
EPHB6: Ephrin type-B receptor 6 precursor
AKT3: AKT Serine/Threonine Kinase 3
SRC: Proto-oncogene tyrosine-protein kinase Sarcoma
YES: Cellular homolog of the Yamaguchi sarcoma virus oncogene
mTOR: The mechanistic target of rapamycin
JAK2: Janus kinase 2
MET: Hepatocyte growth factor receptor
FGFR2: Fibroblast Growth Factor Receptor 4
Raf: Raf-1 proto-oncogene, serine/threonine kinase
FGFR4: Fibroblast Growth Factor Receptor 4
ROR2: Receptor Tyrosine Kinase Like Orphan Receptor 2
ERBB4: Erb-B2 Receptor Tyrosine Kinase 4
ERBB2: Erb-B2 Receptor Tyrosine Kinase 2
