# Supplementary material for: Status of kinases in Epstein-Barr virus and Helicobacter pylori Coinfection in gastric Cancer cells
Source: BMC Cancer. 2020 Sep 29;20:925. doi: 10.1186/s12885-020-07377-0 (PMC7523314; doi:10.1186/s12885-020-07377-0)
Supplement: Supplementary file 1 — Additional file 1. [file 12885_2020_7377_MOESM1_ESM.pdf]

Supporting Information

Status of kinases in Epstein-Barr virus and *Helicobacter pylori* Coinfection in Gastric Cancer Cells

Charu Sonkar[1], Tarun Verma[1], Debi Chatterji[2], Ajay Kumar Jain[2] and Hem Chandra Jha[1]**

[1] The discipline of Biosciences and Biomedical Engineering, Indian Institute of Technology Indore, Khandwa Road, Simrol, Indore 453552, India

[2] Choithram Hospital and Research Centre Indore, Indore, Madhya Pradesh, India

****Corresponding Author**

Dr. Hem Chandra Jha

Discipline of BSBE,

Room no. 302, School Building, IIT Indore, Simrol

Phone: +91 9971653189

Email: hemcjha@iiti.ac.in

**Content**	**Page No.**
**Figure S1: Gram's staining of *H. pylori* strain**	S-3
**Figure S2: Genomic DNA isolation for samples**	S-3
**Figure S3: 16s PCR product of samples**	S-3
**Figure S4: *H. pylori* and EBV co-infection leads to morphological changes**	S-4
**Figure S5: *H. pylori*-infected AGS cells stained with DAPI**	S-4
**Figure S6: Cell length measurement at 12 h**	S-4
**Figure S7: 24 hrs without insert**	S-5
**Figure S8: 36 hrs without insert**	S-6
**Figure S9: 24 hrs with insert**	S-7
**Figure S10: 36 hrs with insert**	S-8

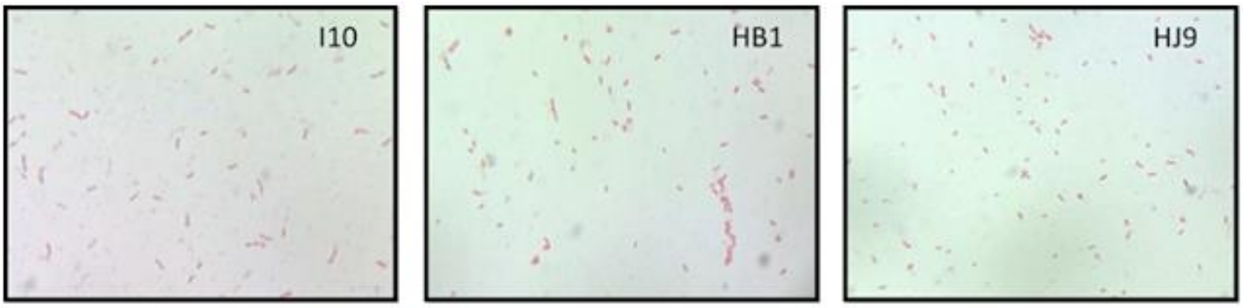

Figure S1. Gram's staining of *H. pylori* strain: Gram's staining of *H. pylori* strain I10 and isolates HB1, HJ9 respectively

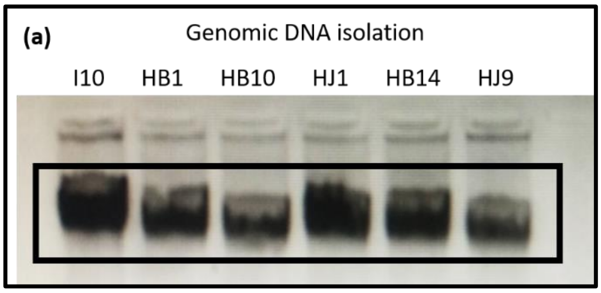

Figure S2. Genomic DNA isolation for samples: *H. pylori* isolates were obtained through genomic isolation for samples I10, HB1, HB10, HJ1, HB14, and HJ9.

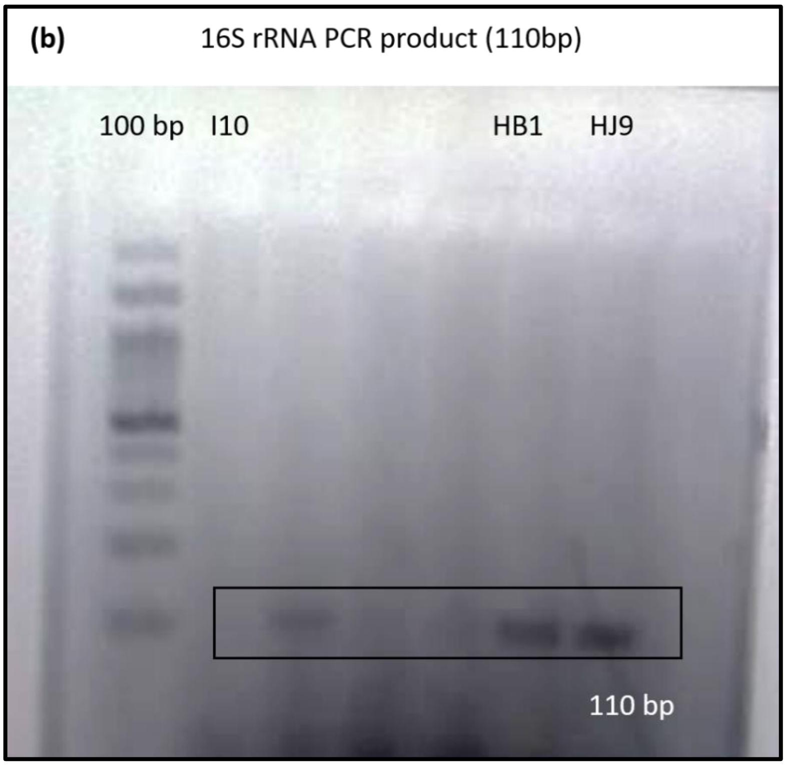

Figure S3. 16s PCR product of samples: 16s PCR product size of 110bp was obtained for I10, HB1, and HJ9, respectively.

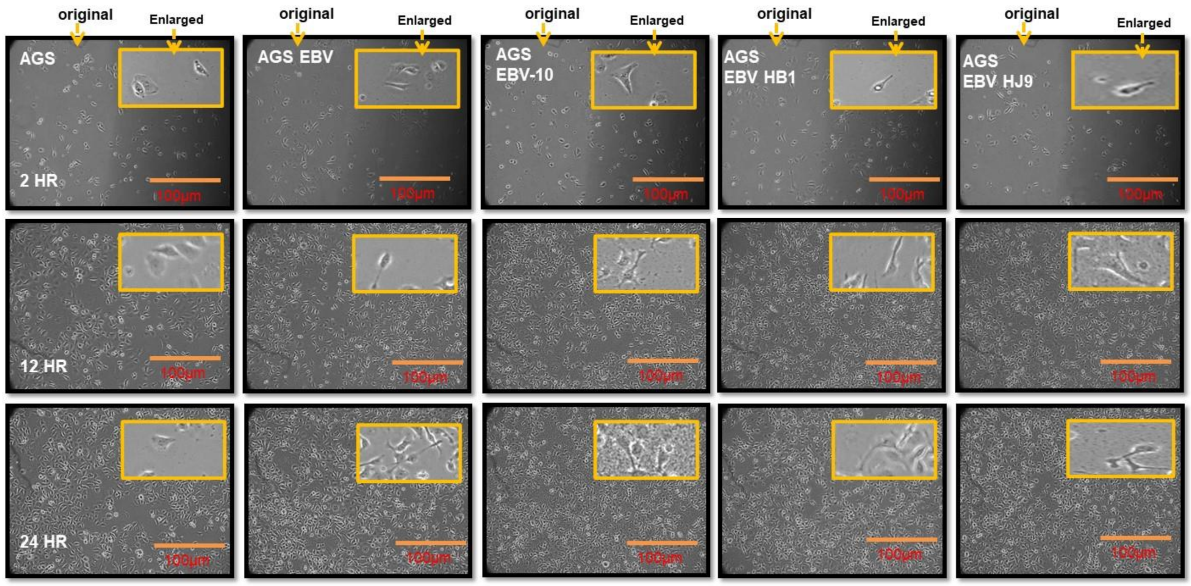

Figure S4. ***H. pylori*** **and EBV co-infection leads to morphological changes:** AGS cells were infected with EBV and then AGS cells were infected with EBV and *H.pylori* I10, HB1, HJ9 respectively. Changes in number of cells and morphological changes were observed at 2h, 12 h and 24 h where insert image shows the enlarged image of morphological changes**.**

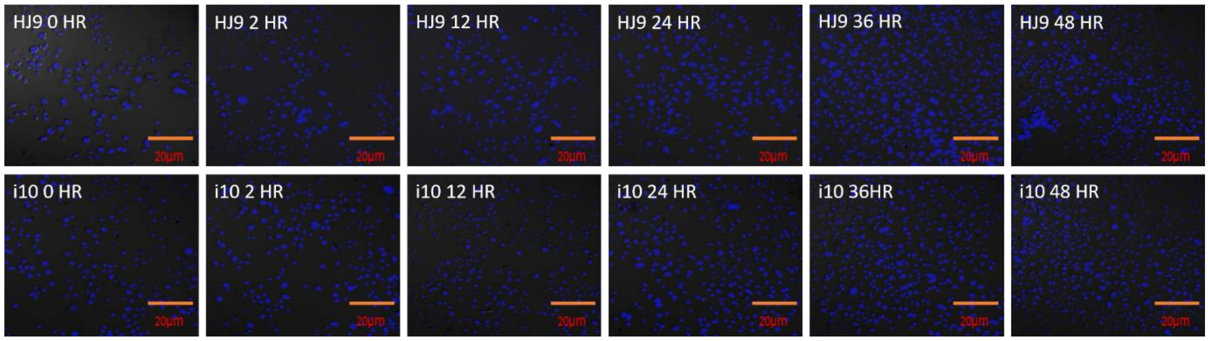

Figure S5. ***H. pylori*****-infected AGS cells stained with DAPI:** AGS cells were infected with I10, HJ9, and stained with DAPI and at 0, 2, 12, 24, 36 and 48 h cell length was measured through ImageJ software.

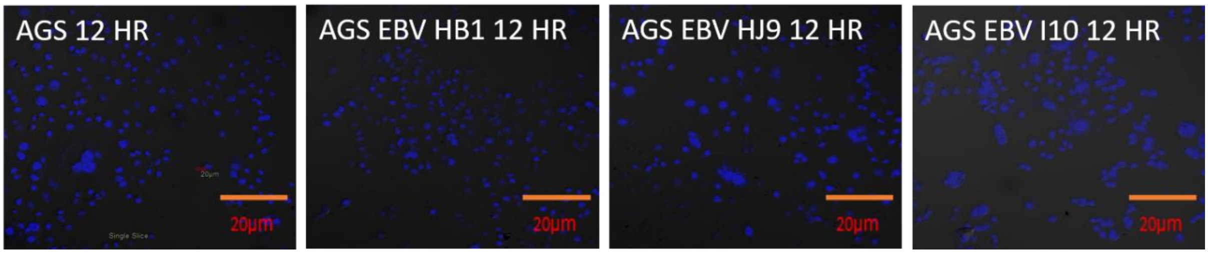

Figure S6. Cell length measurement at 12 h: AGS cells were then infected with I10, HJ9 followed by infection with EBV in each infected cell, and cell length was measured at 12 h through image J software.

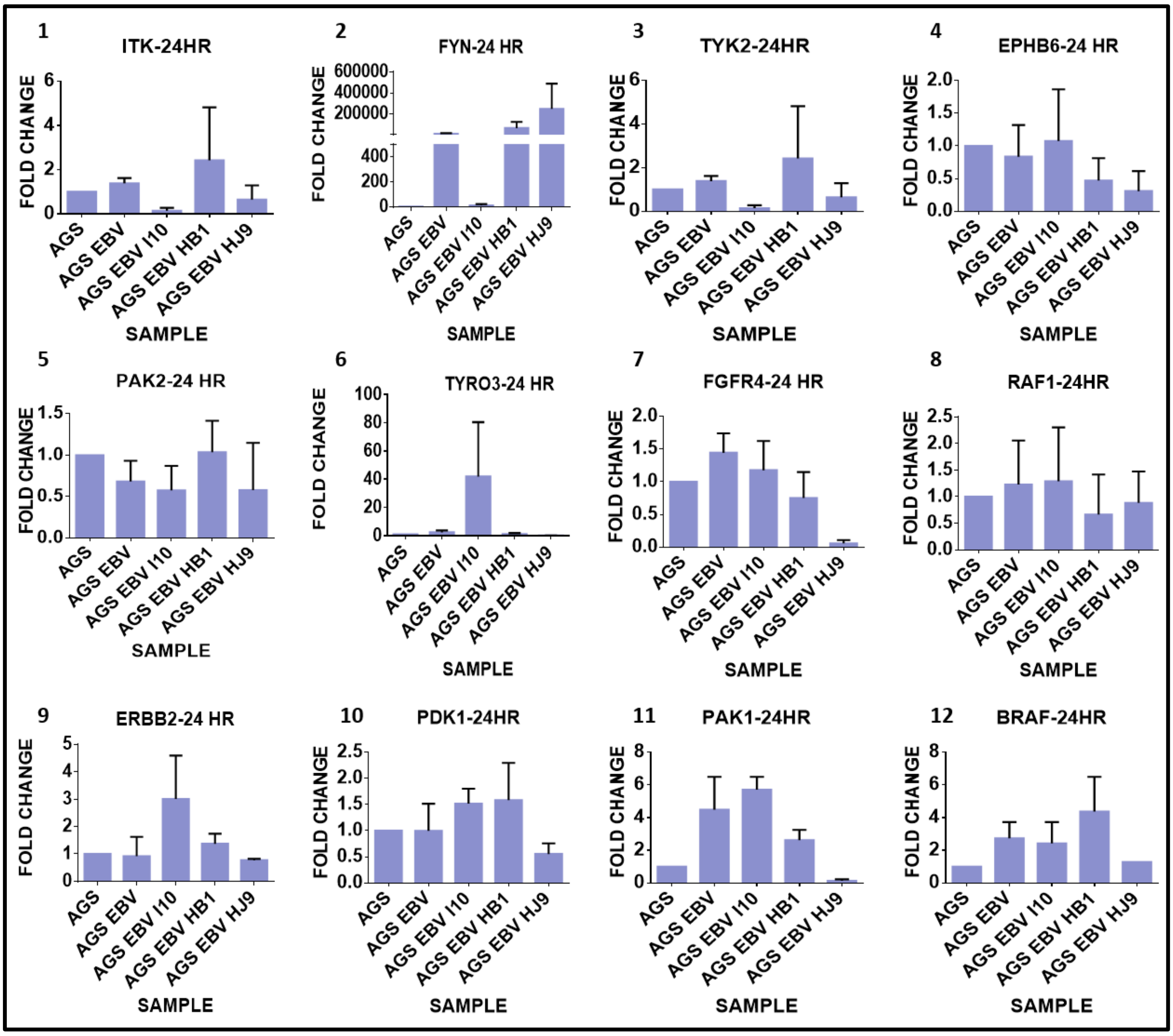

Figure S7. 24 hrs without insert: Assessment of kinase gene's expression was shown with a direct approach at 24 hr.

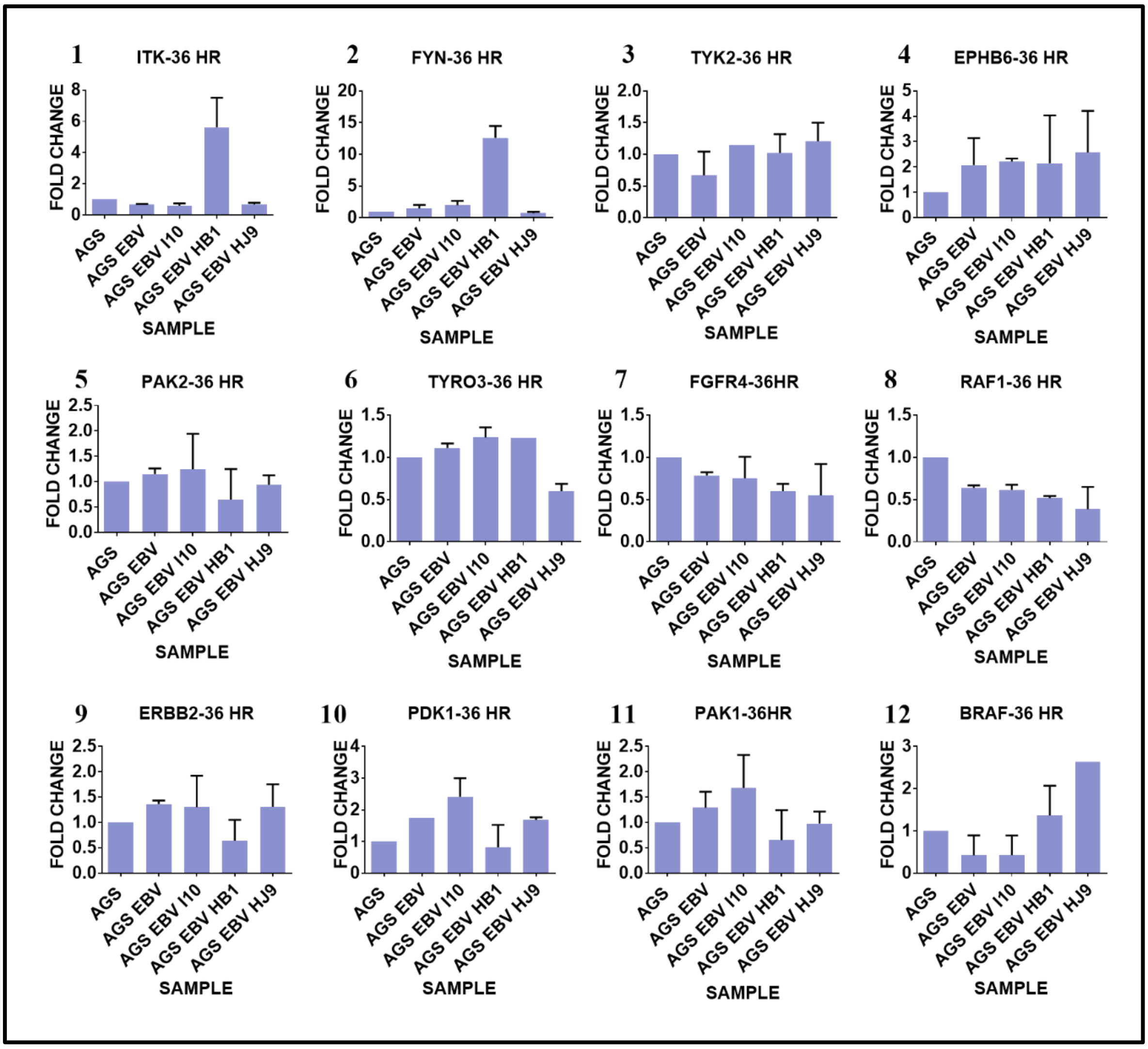

Figure S8. 36 hrs without insert: Assessment of kinase gene's expression was shown with a direct approach at 36 hr.

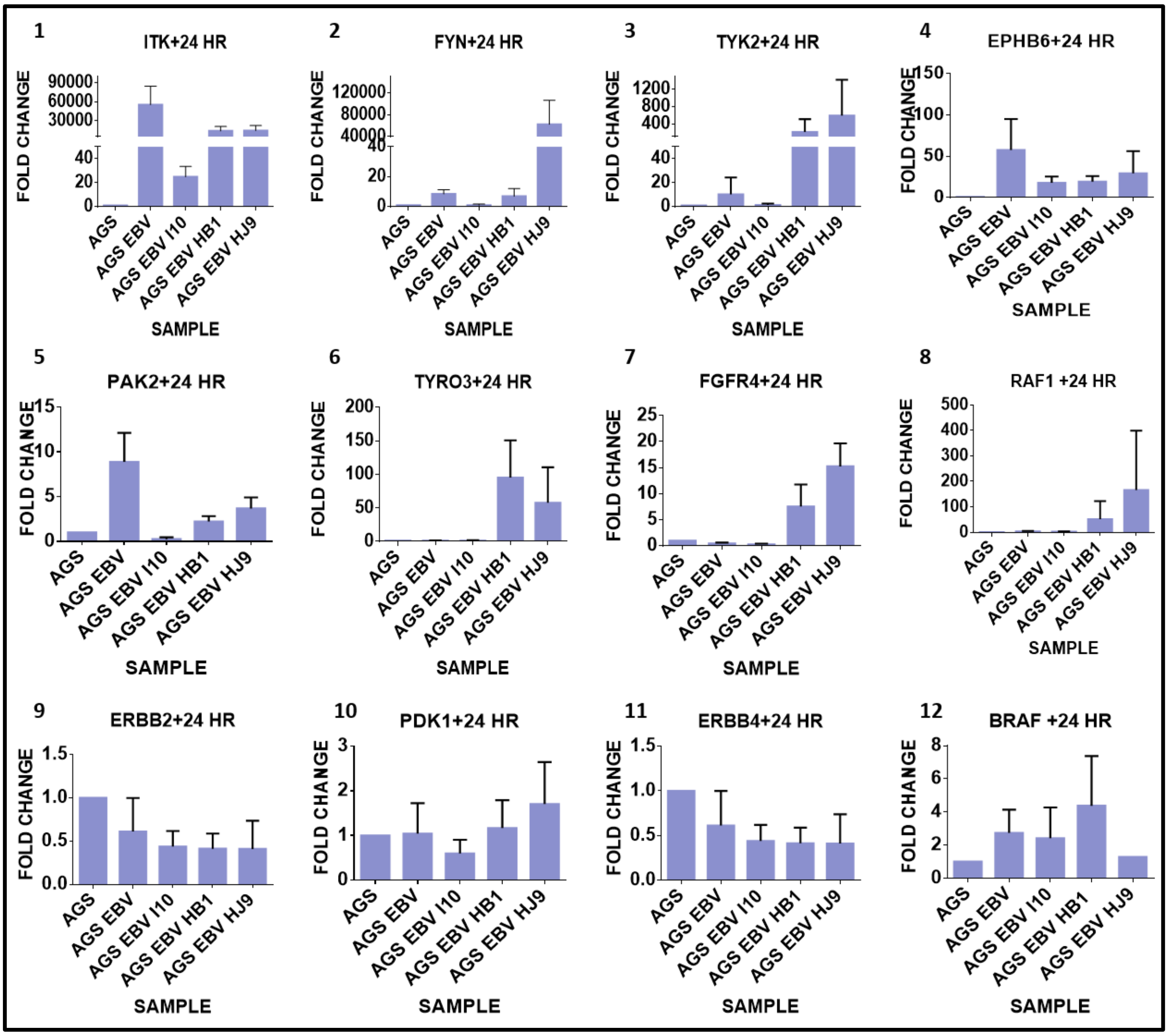

Figure S9. 24 hrs with insert: Assessment of kinase gene's expression was shown with an indirect approach at 24 hr.

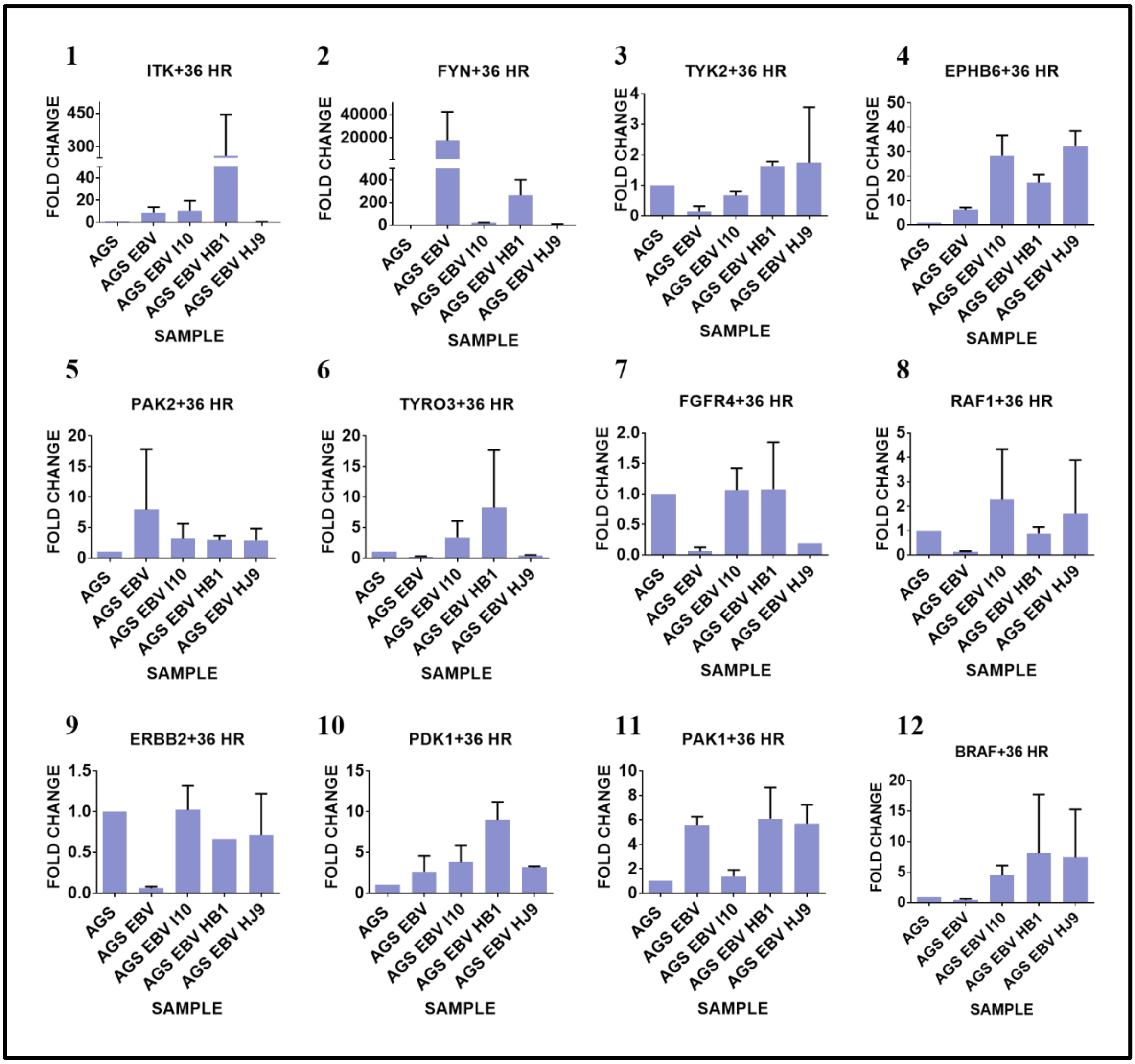

Figure S10. 36 hrs with insert: Assessment of kinase gene's expression was shown with an indirect approach at 36 hr.
